# Stimulus-specific behavioral responses of zebrafish to a large range of odors exhibit individual variability

**DOI:** 10.1186/s12915-020-00801-8

**Published:** 2020-06-15

**Authors:** Florence Kermen, Lea Darnet, Christoph Wiest, Fabrizio Palumbo, Jack Bechert, Ozge Uslu, Emre Yaksi

**Affiliations:** 1 Kavli Institute for Systems Neuroscience and Centre for Neural Computation, Faculty of Medicine and Health Sciences, Norwegian University of Science and Technology, 7030 Trondheim, Norway; 2 Neuro-Electronics Research Flanders, Imec Campus, Kapeldreef 75, 3001 Leuven, Belgium; 3 Department of Biology, Faculty of Natural Sciences, Norwegian University of Science and Technology, 7491 Trondheim, Norway; 4 KU Leuven, 3000 Leuven, Belgium; 5 Medical Research Council Brain Network Dynamics Unit, Nuffield Department of Clinical Neurosciences, University of Oxford, Oxford, OX3 9DU UK

**Keywords:** Olfaction, Olfactory ethogram, Odor valence, Zebrafish, Behavior, Blood, Alarm odor, Defensive behavior, Individual variability, Calcium imaging

## Abstract

**Background:**

Odor-driven behaviors such as feeding, mating, and predator avoidance are crucial for animal survival. The neural pathways processing these behaviors have been well characterized in a number of species, and involve the activity of diverse brain regions following stimulation of the olfactory bulb by specific odors. However, while the zebrafish olfactory circuitry is well understood, a comprehensive characterization linking odor-driven behaviors to specific odors is needed to better relate olfactory computations to animal responses.

**Results:**

Here, we used a medium-throughput setup to measure the swimming trajectories of 10 zebrafish in response to 17 ecologically relevant odors. By selecting appropriate locomotor metrics, we constructed ethograms systematically describing odor-induced changes in the swimming trajectory. We found that adult zebrafish reacted to most odorants using different behavioral programs and that a combination of a few relevant behavioral metrics enabled us to capture most of the variance in these innate odor responses. We observed that individual components of natural food and alarm odors do not elicit the full behavioral response. Finally, we show that zebrafish blood elicits prominent defensive behaviors similar to those evoked by skin extract and activates spatially overlapping olfactory bulb domains.

**Conclusion:**

Altogether, our results highlight a prominent intra- and inter-individual variability in zebrafish odor-driven behaviors and identify a small set of waterborne odors that elicit robust responses. Our behavioral setup and our results will be useful resources for future studies interested in characterizing innate olfactory behaviors in aquatic animals.

## Background

Odors are powerful drivers of a wide range of behavioral responses in fish, such as reproduction, foraging, and defensive behaviors [1–3]. The neural pathways underlying these stereotyped behaviors have been the focus of extensive research and are well described [4–15]. In the sea lampreys, a neural pathway comprising the olfactory bulb, posterior tuberculum, and mesencephalic locomotor region converts olfactory inputs into locomotor outputs by activating conserved brainstem pre-motor neurons (the reticulospinal neurons) [16–19], which in turn control the spinal cord locomotor centers [4]*.* Food-related odorants activate hypothalamic regions involved in appetite control in zebrafish [8] and evoke foraging behavior in a wide range of fish species [7, 8, 20–23]. In the zebrafish, alarm odors activate nuclei located in the dorso-medial (Dm) [13, 24] and ventral (Vv) telencephalon [24], as well as in the preoptic area [24]. Respectively, these zebrafish brain regions are homologous to the mammalian amygdala, septum, and paraventricular nucleus of the hypothalamus involved in adaptive fear response and anti-predatory behaviors [25–28]. Thus, a precise characterization of the link between ecologically relevant odors and odor-driven behaviors is an important step towards characterizing the neural circuits generating these essential behaviors and how they are affected by animal’s internal states, such as hunger, fear, or anxiety. Paradoxically, while the zebrafish olfactory circuitry is well characterized, a comprehensive description of zebrafish behavior in response to ecologically relevant odors is needed, to better relate olfactory computations to animal behavior.

A growing number of studies have begun to address this gap in knowledge by characterizing the change in zebrafish swimming patterns in response to odors, identifying clear negative (avoidance) and positive (approach) chemotactic responses [4, 5, 7–12, 14, 15]. These studies investigated the behavioral responses to odors belonging to one or two of the following categories: food-related odors [7, 8, 21], social-related odors [9, 13, 29], decay-related odors (polyamines in decomposing flesh) [30], and alarm odors [24, 31, 32]. This approach precludes the comparison of behavioral responses between all four odor categories within the same individual that is important to uncover stereotyped and odor-specific motor programs. For example, to our knowledge, the response to aversive decay-related odors and alarm odors was not compared in the same individual. Moreover, odor-driven behaviors were either measured in groups of fish, thus masking potential inter-individual variability in odor sensitivity or preferences [9, 13, 21, 24], or the inter-individual variability was not quantified [7, 8, 30–33]. Therefore, there is an important need for measuring and analyzing the behavioral responses of individual fish to a broad range of odors, spanning the natural stimulus space.

Here, we characterize zebrafish odor-driven behaviors using a medium-throughput setup allowing for exposure to well-defined odor concentrations. Using this approach, the swimming trajectories of 10 fish were recorded in response to 17 ecologically relevant odors. By selecting seven appropriate locomotor metrics, we constructed behavioral ethograms systematically describing odor-induced changes in the swimming trajectory. We found that fish reacted to most odorants with different behavioral programs. A combination of few relevant behavioral metrics was sufficient to capture most of the variance in these innate odor responses. Odors in similar categories elicited weakly clustered behavioral responses. In addition, we quantified intra- and inter-individual variability of odor-driven behaviors and suggest a small set of odors that elicit robust responses. Finally, we showed that conspecific blood and the alarm odor “skin extract” elicit very similar defensive behaviors and activate overlapping regions in the dorso-lateral olfactory bulb.

## Results

### A vertical olfactory setup with precise control of odor concentration and fast switching of odors

**Fig. 1.**
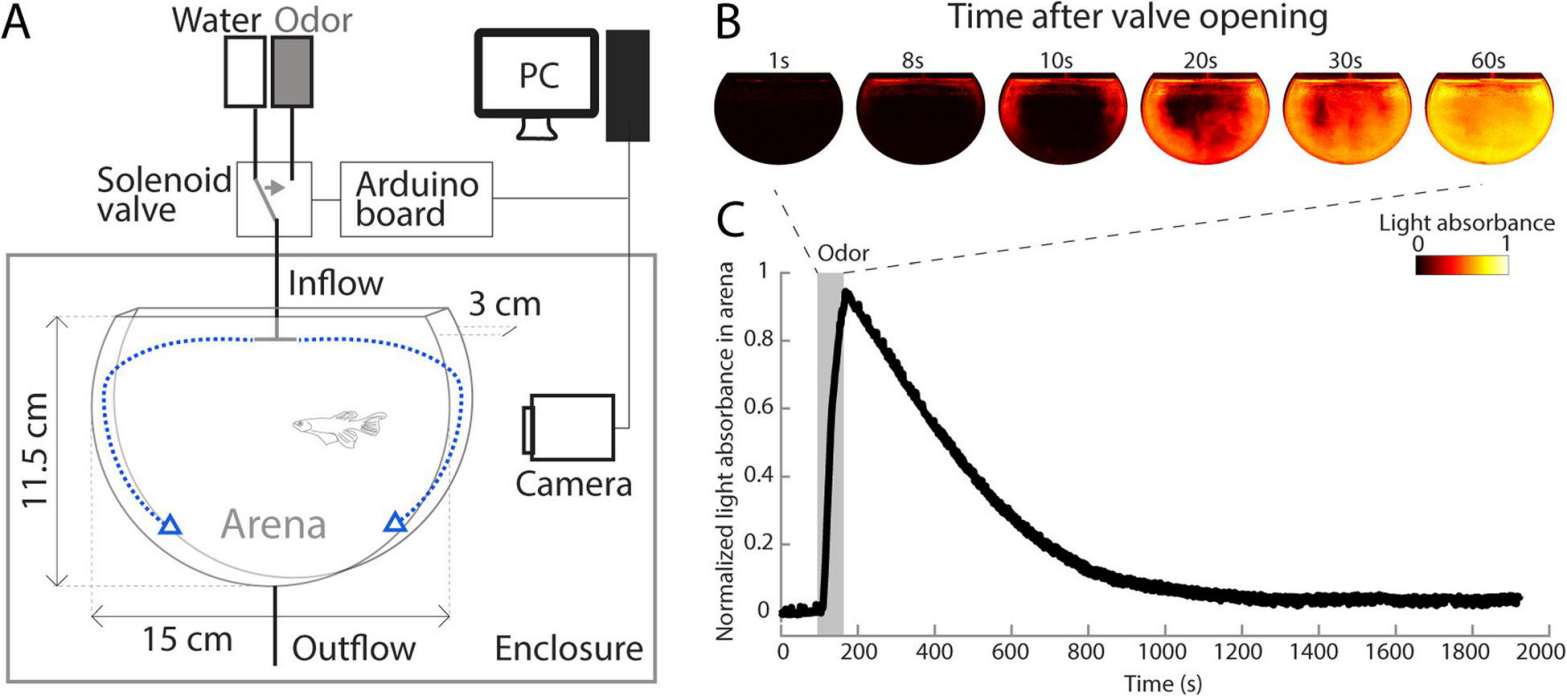
Design of the behavioral setup. **a** Schematic of the behavioral setup enabling the rapid delivery of odors to the arena using a computer-controlled solenoid valve. The arena dimensions were as follows: horizontal axis = 15 cm, vertical axis = 11.5 cm, and width = 3 cm. The schematic of the fish is to scale with the arena. The blue dashed arrows indicate the water flow deflection on the walls of the arena. **b** Image series showing the rapid and evenly spread of the odor within the arena after valve opening. The odor was replaced by a dye, and its normalized concentration in the arena is plotted over time (0 = no stimulus; 1 = same concentration as in the odor bottle). **c** Dynamic of stimulus concentration in the arena over the course of 30 min. The valve was open for 1 min

To reproducibly measure fish responses to a large variety of odorants, we built a computer-controlled setup automatically recording the position of freely swimming individual fish (Fig. 1a). The arena contained ~ 400 mL of water and measured 15 cm along the horizontal axis, 11.5 cm along the vertical axis, and was 3 cm wide (approximately six by five by one fish body lengths), which allowed us to investigate zebrafish displacement in both the vertical and horizontal dimensions. The flow rate was adjusted to 90 mL/min, which was fast enough to rapidly clear the arena, but not strong enough to exhaust or stress the fish. In addition, a T-shaped connector deflected the inflow towards the lateral walls (Fig. 1a). This was to avoid pushing the fish down and to enable a rapid and homogeneous distribution of the stimuli within the arena. To characterize the onset and dynamic of the odor delivered to the arena, we replaced it by a dye and measured the change in reflected light over time (Fig. 1b). The dye reached the arena 8 s after the valve opened and rapidly spread through the arena, covering its entire volume within 30 s. The dye concentration had returned to pre-stimulus levels within 15 min (Fig. 1c). Based on this, we chose an inter-trial duration of 20 min to ensure complete clearance of prior odors before the following recording started.

### Characterization of zebrafish behavior in response to diverse odors

Fish rely on different categories of water-soluble odors to guide behaviors essential for survival. We thus chose ecologically relevant odors related to the four following categories: feeding, social, decay, and alarm odors (Additional file 1: Table S1). We used seven different feeding-related odorants: five amino acids, which are substances eliciting foraging [7]; a mix of nucleotides signaling the freshness of food [8]; and food components extracted from fish-food flakes. Social odorants consisted of commercially available chemicals that were shown to be present in fish excretions: a mixture of bile acids, which guide sea lampreys to spawning sites [34]; ammonium and urea, which are metabolites present in fish urine [35]; and prostaglandin 2α, a fish reproductive pheromone released by ovulating females [9]. Odors signaling decay consisted of putrescine, cadaverine, and spermine, which are three amines enriched in decaying flesh and avoided by zebrafish [30]. Finally, alarm odors consisted of zebrafish skin extract [36]; chondroitin sulfate, a component of fish skin extract that elicits alarm response in zebrafish [31]; and zebrafish blood, a putative alarm odor, eliciting defensive behaviors in tilapia [28].

**Fig. 2.**
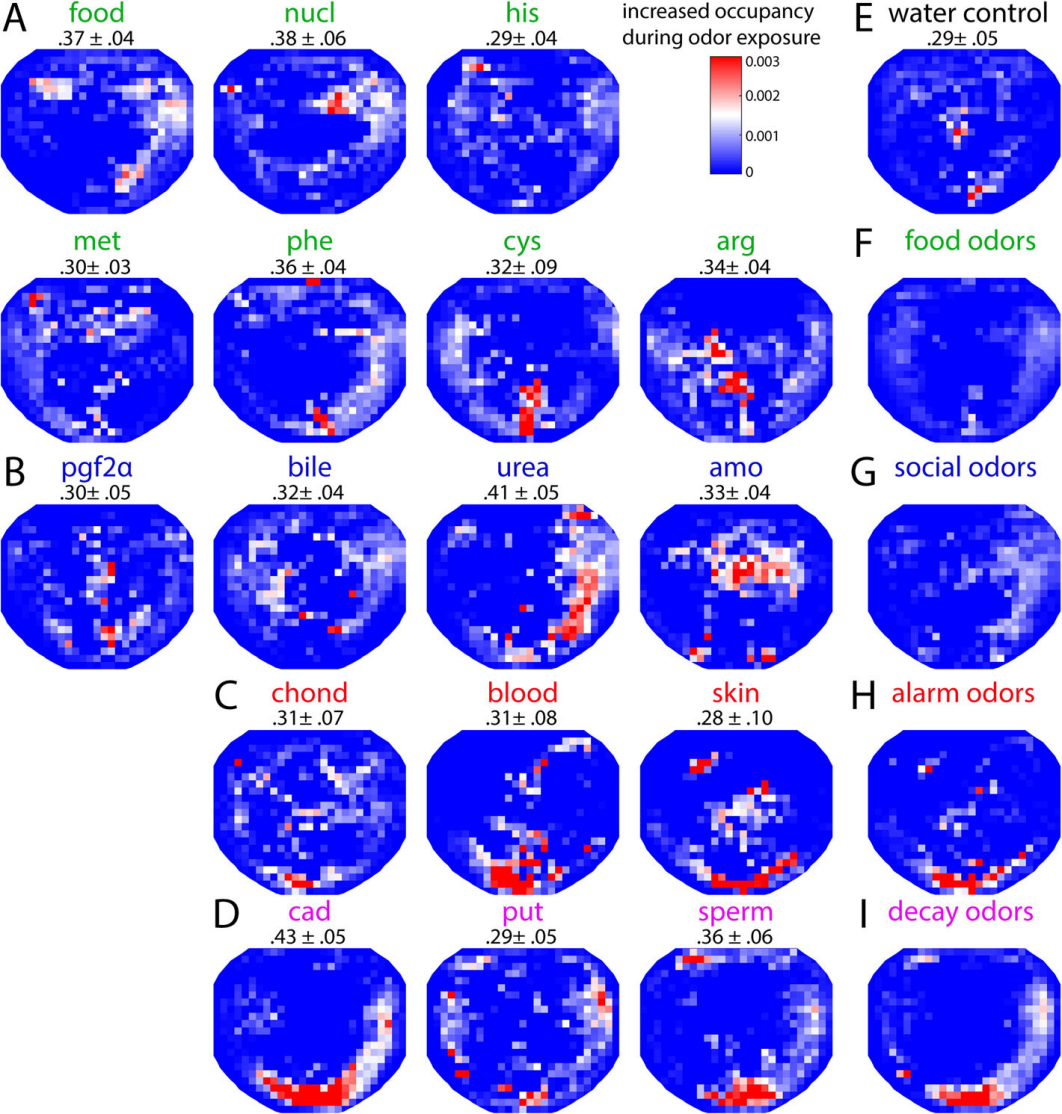
Spatial response of adult zebrafish to ecologically relevant odorants. Average occupancy maps representing the swim location of zebrafish during odor exposure (*n* = 10 zebrafish, see the “Methods” section) in response to **a** food-related odors in green (food extract, nucleotides, histidine, methionine, phenylalanine, cysteine, and arginine), **b** social-related odors in blue (prostaglandin 2α, bile acids, urea, and ammonium), **c** decay odors in magenta (putrescine, spermine, cadaverine), **d** alarm odors in red (chondroitin sulfate, zebrafish blood, zebrafish skin extract), and **e** water control in black. The numbers located under the odor label for maps in **a**–**e** refer to the spatial correlation (mean ± SEM) of individual maps to the average occupancy map (spatial stereotypy). **f** Average of all food odor maps in **a**. **g** Average of all social odor maps in **b**. **h** Average of all alarm odor maps in **c**. **i** Average of all decay odor maps in **d**


To characterize zebrafish odor-driven behaviors, we then measured the swimming trajectory of 10 adult fish (seven males and three females) in response to these 17 odorants and to a water control (Additional file 2: Data S1). Single fish were habituated to the arena for 45 min at the beginning of a daily recording session. During each individual recording lasting 10 min, an odor was delivered after an initial period of 5 min without odor. Mapping the fish position yielded average occupancy maps representing the swim location of zebrafish during odor exposure (Fig. 2a–d) that differed markedly across odorants. In particular, feeding cues such as food extract, nucleotides, and methionine induced exploration of the upper part of the arena (Fig. 2a, Additional file 3: Movie S1), where the odor was first delivered. In contrast, fish swam at the bottom of the tank in response to alarm odors such as blood and skin extract (Fig. 2c, Additional file 4: Movie S2). To examine whether some odors evoked more stereotypic displacements than others, we quantified the average similarity of occupancy maps for individual fish with the group average, for each odor (Fig. 2a–d). The relatively high spatial stereotypy values indicate that these average occupancy maps reflect the contribution of individual fish. We found no difference in spatial stereotypy between odors (one-way ANOVA, *F*(17, 154) = 0.57; *p* = 0.910).

**Additional file 3:Movie S1.** Response of a fish to food odor. A representative example of zebrafish appetitive response to food odor. Upon odor detection, the fish investigates the very top of the arena with abrupt changes in direction. Note that attempts at biting are also visible.



**Additional file 4:Movie S2.** Response of a fish to blood odor. A representative example of zebrafish defensive behavior in response to blood odor. Upon odor detection, the fish first display bursts of high velocity swimming (erratic swimming) throughout the arena, before diving at the bottom and freezing.


Overall, except increased activity closer to the lateral walls, the average occupancy map in response to feeding (Fig. 2f) and social odors (Fig. 2g) showed no clear differences compared to the water control (Fig. 2e). This was likely due to the important inter-odor variability within these categories. Average occupancy maps in response to alarm odors (Fig. 2h) revealed a consistent increase in bottom diving that was also observable, although to a lesser extent, in response to decay odors (Fig. 2i).

### Quantification of odor responses by using behavioral metrics

**Fig. 3.**
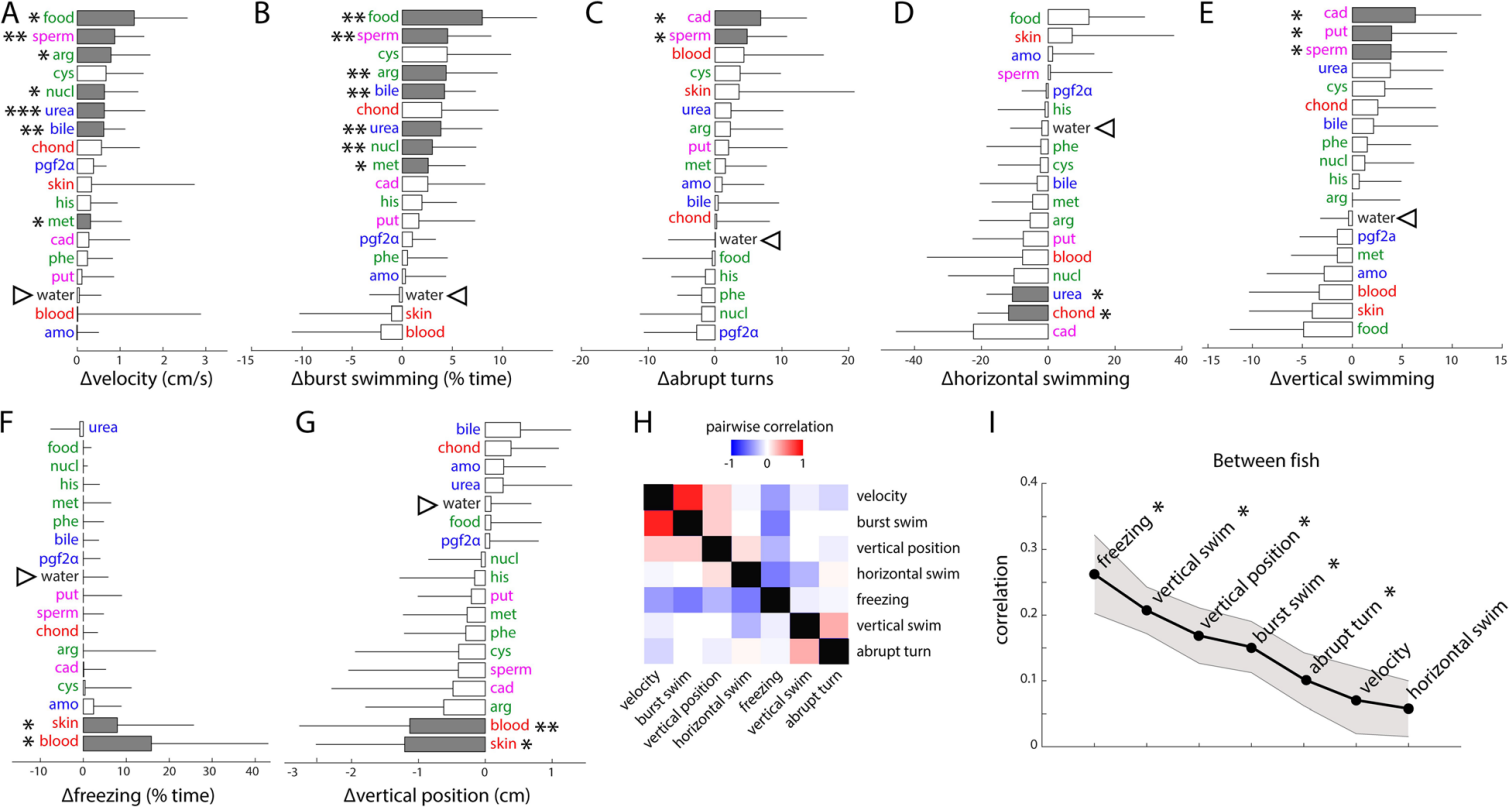
Metrics for quantifying behavioral responses of adult zebrafish to ecologically relevant odorants. Change in seven behavioral response metrics during the first 2 min following odor delivery (Additional file 6**:** Data S2). **a** Velocity, **b** percentage of burst swimming, **c** number of abrupt turns, **d** horizontal swimming, **e** vertical swimming, **f** freezing, and **g** vertical position in the arena. The filled gray bars indicate significant differences from the water control, indicated by an arrowhead (Mann-Whitney *U* test; *p < 0.05, **p < 0.01, and ***p < 0.001, see Additional file 8: Table S2 for all *p* values). Data are represented as median ± standard deviation deviation. Odors are sorted according to their average response for a given metric. Food-related odors are in green, social-related odors in blue, decay odors in magenta, and alarm odors in red. **e** Water control in black. For convenience, a version of the histograms in **a**–**g** without resorting is also available in Additional file 7: Fig. S2. **h** Pairwise correlation (see the “Methods” section) between all behavioral metrics in **a**–**g**. **i** Variability of behavioral metrics across different fish. The average correlation (black line) between the average odor responses of all fish is plotted for all metrics. High correlations indicate low variability. The gray shaded area indicates SEM. Significant differences from the null distribution are indicated by the asterisk symbol (*) (permutation test, see the “Methods” section)

To quantify the dynamics of zebrafish locomotor behavior in response to odors, we calculated the odor-induced changes in the fish velocity (Fig. 3a), the amount of burst swimming (Fig. 3b), and the number of abrupt turns (Fig. 3c), as well as the amount of horizontal (Fig. 3d) and vertical swimming events (Fig. 3e). To quantify the valence of odors, we also calculated the change in metrics for decreased exploration, such as time spent freezing (Fig. 3f) and the position along the vertical axis (Fig. 3g), which reflects fear and defensive behaviors in zebrafish [31, 37]. Representative responses of an individual fish to selected odors are illustrated in Additional file 5: Fig. S1. Using these metrics, we built behavioral ethograms systematically describing the odor-induced changes in response to our diverse set of odors (Fig. 3a–g, Additional file 6: Data S2, Additional file 7: Fig. S2). Importantly, we found no change in any of the behavioral metrics after water control delivery, indicating that small variation in flow rate or vibrations due to the valve opening and closing did not trigger behavioral responses.

We observed significant changes in swimming speed characterized by increased velocity and number of burst swimming events, for four food odors, two social odors, and spermine. A significant increase in the amount of abrupt turns, possibly indicative of erratic swimming [38], was prominent in response to two decay odors (cadaverine and spermine). Interestingly, blood also elicited an increase in sharp turns (Additional file 8: Table S2, *p* = 0.076). Changes in swimming strategy, in particular the amount of horizontal and vertical swimming, have been observed in several fish species, related to foraging and spawning [39]. Here, we observed a decrease in time spent swimming horizontally in response to urea and chondroitin. Interestingly, food extract elicited an increase in the amount of horizontal swimming, a result reminiscent of foraging behaviors. Vertical swimming, up or down, was significantly increased in response to the three decay odors cadaverine, putrescine, and spermine. Remarkably, the metrics related to defensive behaviors (freezing and vertical position in the tank) were significantly modulated solely during exposure to two out of seventeen odors: skin extract and blood. The time spent freezing and time spent at the bottom of the arena increased in response to blood, similar to what has been reported in response to skin extract [25, 31, 38]. Hence, conspecific blood elicits the whole suite of alarm behaviors displayed in response to skin extract and thus is a novel and equally powerful alarm odor in zebrafish.

To determine whether our array of behavioral metrics captured independent aspects of the odor responses, we calculated their average pairwise correlation during odor response (Fig. 3h). As expected, freezing was negatively correlated to most active locomotion metrics (velocity, burst swimming, and horizontal swimming, Fig. 3h) and to the vertical position, which reflects that the majority of freezing took place at the bottom of the arena. With the exception of burst swimming that was strongly correlated to velocity, most metrics exhibited relatively weak correlations, indicating that they captured independent aspects of the odor-induced behaviors.

To determine which behavioral metrics were most stereotyped across fish, we calculated the pairwise correlations between odor response profiles across fish for each metric (Fig. 3i). Most metrics were significantly correlated across fish. In particular, the most stereotyped responses elicited by our set of odors were freezing, vertical swimming, and vertical position in the tank.

### Variability of odor responses within and between individual zebrafish

**Fig. 4.**
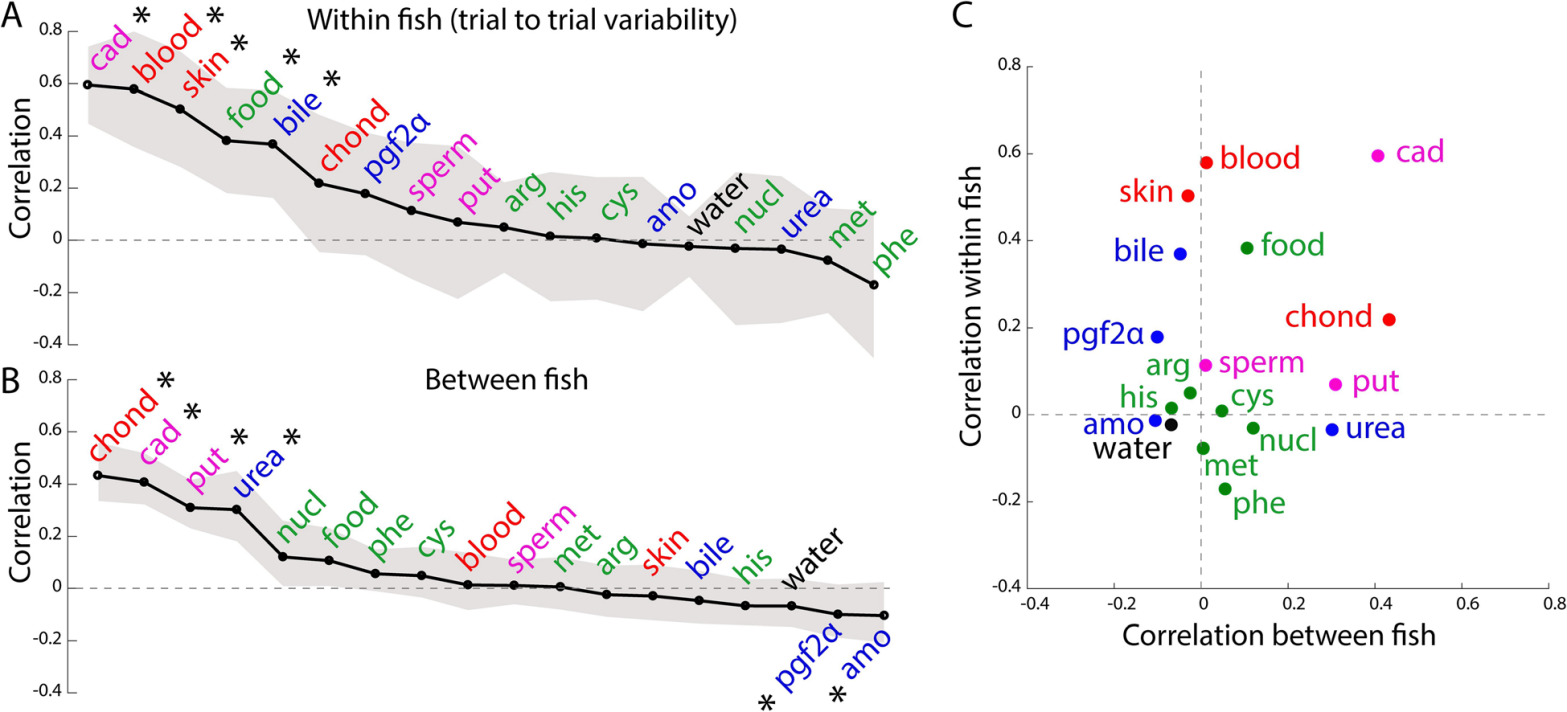
Variability of odor responses within and between individual zebrafish. **a** The average correlation (black line) between odor responses across trials within an individual fish is plotted for all odors. High correlations indicate low variability. Odor responses were represented by a vector of the seven behavioral metrics described in Fig. 3, averaged during the first 2 min of the odor delivery period. **b** Variability of odor responses across different fish. The average correlation (black line) between the average odor responses of all fish is plotted for all odors. The gray shaded area indicates SEM. **c** Summary of data in **a** and **b**. Food-related odors are in green, social-related odors in blue, decay odors in magenta, and alarm odors in red. Significant differences from the null distribution are indicated by the asterisk symbol (*) (permutation test, see the “Methods” section)

The variability of behavior within and across individuals is an important aspect of an animal’s response [40] that quantifies how reproducible the observable behavior is over repeated presentations. During our experiments, we observed variable responses between individual zebrafish. For example, one fish displayed behavioral responses that differed from that of the majority of other fish (Additional file 9: Fig. S3). Despite such variability across individuals, the trial to trial variability of an individual’s responses to different presentations of the same odor is rather low, indicating high reproducibilty (Additional file 5: Fig. S1). For example, the patterns of freezing and vertical position in response to blood and the number of abrupt turns in response to skin extract can be remarkably similar across replicates (Additional file 5: Fig. S1A,C,G). However, this is not the case for all odors, indicating that only a few specific odors might generate reproducible behaviors within fish. In order to quantify whether odor responses were reproducible across trials, we measured the average correlation between behavioral responses to odor replicates (Fig. 4a). Most odors elicited weakly correlated responses. However, cadaverine, blood, skin extract, food odor, and bile acids displayed higher correlations across trials that ranged from 0.37 to 0.59 (Fig. 4a), which confirmed that specific subsets of odors can elicit reproducible responses within individual fish. In order to determine whether odors elicit similar responses across different fish, we calculated the average correlation of individual odor responses between all pairs of fish (Fig. 4b). We observed that chondroitin sulfate, cadaverine, putrescine, and urea elicited the most reproducible responses across fish (Fig. 4b). Interestingly, cadaverine elicited reproducible responses both within and between fish (Fig. 4c).

### Categorization of odors based on behavioral metrics

**Fig. 5.**
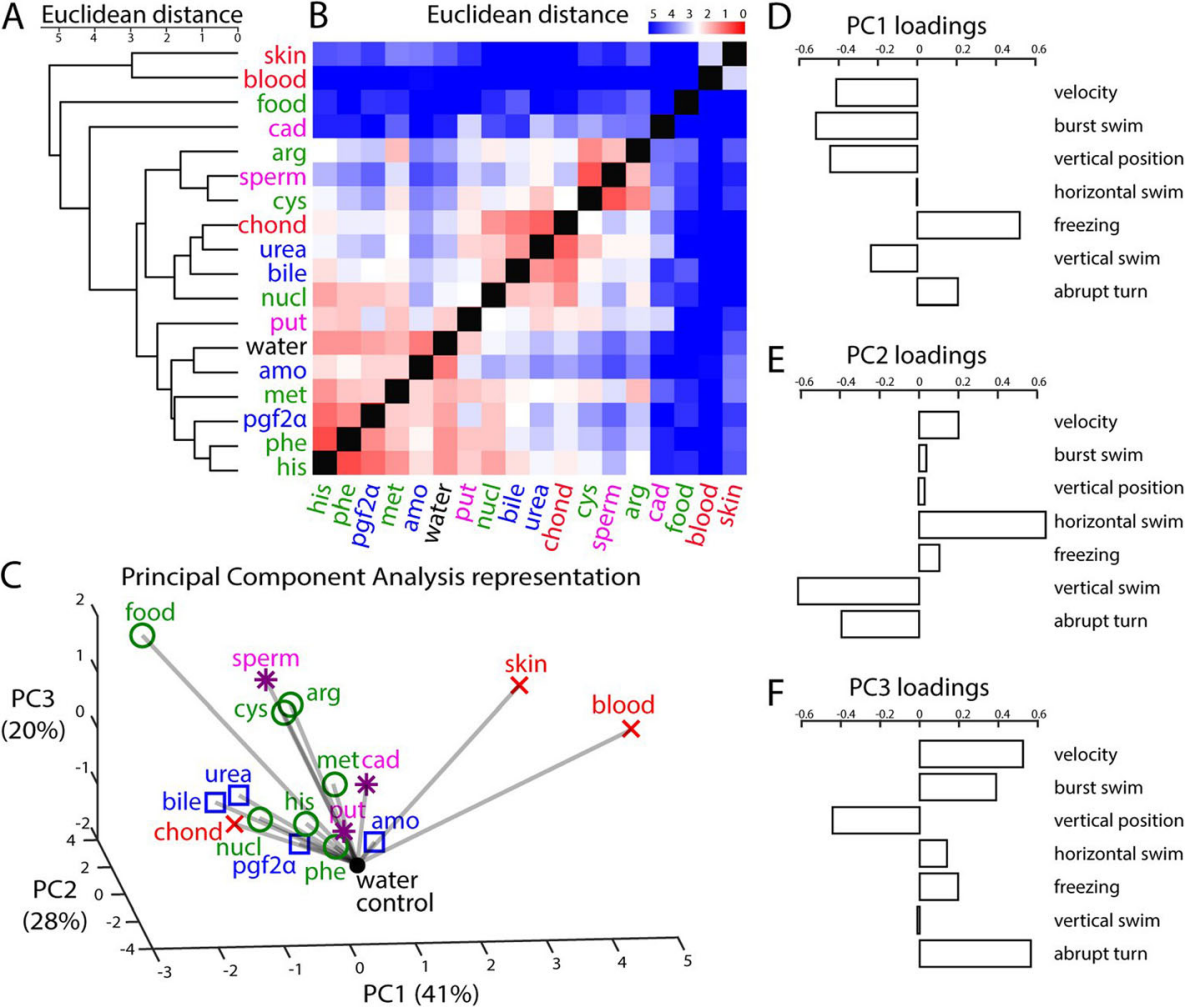
Behavior-based categorization of odors. **a** Dendrogram representing the hierarchical clustering based on Euclidean distances between odor-driven behaviors. **b** Pairwise Euclidean distances between behavioral responses to all odors using seven behavioral metrics (*n* = 10 fish; see the “Methods” section). Red indicates similar, and blue dissimilar, behavioral responses. **c** Representation of all odors with the first three principal components (PCs, see the “Methods” section). The percentage of behavioral variance explained by each PC is indicated between brackets. Gray lines indicate the distance of each odor to the water control (filled black dot). Food-related odors (food extract, histidine, nucleotides, methionine, phenylalanine, cysteine, and arginine) are represented by green circles. Social-related odors (bile acids, prostaglandin 2α, urea, and ammonium) are represented by blue squares. Decay odors (putrescine, spermine, cadaverine) are represented by magenta asterisks. Alarm odors (chondroitin sulfate, zebrafish blood, zebrafish skin extract) are represented by red crosses. **d**–**f** Respective contribution of all seven behavioral metrics to the three first principal components (PC1, PC2, and PC3)

Next, we set to categorize odors based on behavioral responses, using a combination of all the behavioral metrics described above. To do this, we used hierarchical clustering to group odors eliciting similar behavioral responses (Fig. 5a), based on the Euclidean distances between all pairs of odors (Fig. 5b). Clustering our broad set of odors based on zebrafish behavioral responses led to a categorization of odors. A subset of amino acids, composed of methionine, histidine, and phenylalanine, did not elicit responses prominently different from water control, likely due to the partial contribution of these individual components of food extract to feeding behavior. A second subset of amino acids, composed of cysteine and arginine, clustered together with the decay-related amine spermine. A third subset, composed solely of the natural feeding odor, food extract, elicited a different behavioral response from its amino acid components. Similarly, although two of the alarm odors (blood and skin extracts) clustered together and were clearly different from all other odorants, the monomolecular alarm odor chondroitin sulfate was not part of that cluster.

To further facilitate the visualization of the complex behavioral response to the different odor categories, and to account for the dependencies between response measures (Fig. 3h), we performed a dimensionality reduction analysis using principal component analysis (PCA, Fig. 5c). We found that the first three principal components (PCs) of the behavioral space accounted for 89% of the variance, suggesting that olfactory responses can be explained by a combination of a few major behavioral programs. The PC1 clearly segregated food extract at one end and blood as well as skin extract at the other end (Fig. 5c; Additional file 10: Fig. S4A,C). PC1 was positively correlated with decreased locomotion (velocity, burst swim) and increased defensive behavior indices (bottom diving and freezing (Fig. 5d)) thus possibly contrasting immediate rewards and threats. PC2 mostly separated animals’ movement in the horizontal versus vertical dimensions (Fig. 5e), which is likely to represent defensive versus exploratory behaviors. Finally, PC3 mostly represented parameters related to locomotion speed (Fig. 5f). Taken together, our results suggest that zebrafish odor-driven behaviors in response to 17 odors can be explained by a handful of behavioral metrics represented by the first three PCs.

### The alarm odors, skin extract and conspecific blood, activate spatially overlapping olfactory bulb domains

**Fig. 6.**
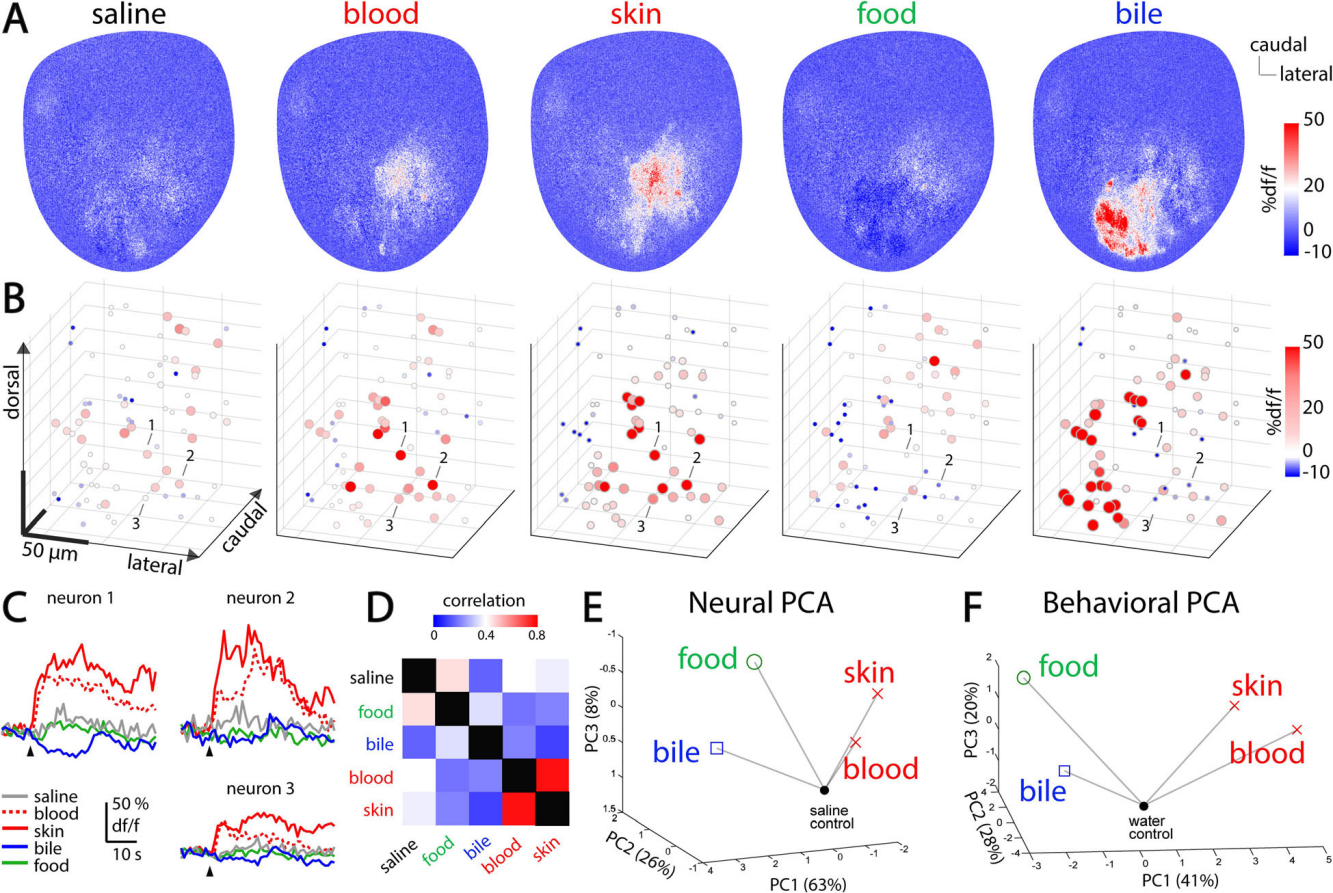
Zebrafish blood and skin extract elicit highly similar neural activity in the dorsal olfactory bulb. Olfactory bulb responses to zebrafish blood, zebrafish skin extract, food odor, bile acid, and saline control (artificial cerebrospinal fluid) were measured using two-photon calcium imaging in adult zebrafish brain explants (*n* = 4 fish; see the “Methods” section). **a** Calcium signals representing the odor responses averaged across all optical planes in a representative fish. Warm colors represent stronger neural activity. **b** Three-dimensional reconstructions of olfactory bulb responses to all odors in the same fish as in **a**. Each circle represents an individual neuron. The spatial orientation and scale bars are indicated on the left graph and are the same for all representations. **c** Odor responses of the three individual neurons selective for skin extract and blood indicated in **a**. **d** Correlation matrix representing the average pairwise similarity between multi-neuronal odor representations (*n* = 4 fish). **e** Representation of all odors with the first three principal components of the neural responses (PC, see the “Methods” section). The percentage of variance explained by each PC is indicated between brackets. Gray lines indicate the distance of each odor to the saline control (filled black dot). **f** Representation of the same odors with the first three principal components of the behavioral responses (adapted from Fig. 5c). The data used in this figure are provided in Additional file 11: Data S3

Our behavioral data suggest that zebrafish can smell blood and exhibit defensive behaviors that are similar to responses induced by the commonly used alarm odor, skin extract. To investigate whether conspecific blood activates the zebrafish olfactory system, we measured olfactory bulb responses to a subset of our odors, using two-photon calcium imaging in a brain explant preparation of Tg(elavl3:GCaMP6s) adult zebrafish [6, 11, 41, 42] (see the “Methods” section). We found that both blood and skin extract specifically activated a subset of olfactory bulb neurons located in the antero-lateral domain, below the dorsal surface of the olfactory bulb (Fig. 6a–c). To quantify the similarities of odor responses, we calculated the pairwise Pearson’s correlations between multi-neuronal representations of these odors. Our analysis showed that blood and skin extract elicited very similar neural responses in the dorsal olfactory bulb that were clearly distinct from those evoked by food extract, bile acid, and the saline control (Fig. 6d). These results revealed for the first time that conspecific blood specifically activates the zebrafish dorsal olfactory bulb with a pattern similar to the commonly used alarm odor, skin extract. Interestingly, odors that elicited different behavioral responses as measured with our set of behavioral metrics (such as food and skin extract) were also segregated based on the distinct neural activation that they evoked in the dorsal olfactory bulb (Fig. 6e, f). In summary, our results revealed that skin extract and blood eliciting similar behavioral responses also exhibit highly similar neural activation patterns.

## Discussion

### Advantages and limitations of the medium-throughput odor-driven behavior assay

We describe a medium-throughput setup to measure the behavior of individual fish exposed to odors of known concentrations. The automated delivery combined with rapid odor clearance enables to test multiple stimuli a day without manipulating the fish, while ensuring trial-to-trial reproducibility in stimulus dynamic. Precise control of odor concentration and dynamic is crucial for reproducible experiments, since it influences odor detection and valence [43]. Unlike studies in terrestrial animals, where the delivery of airborne chemicals is controlled by olfactometers and measured by photoionization detectors [44, 45], measuring and rapidly clearing odors in the turbulent aquatic medium has proven challenging [46]. Therefore, aquatic studies often confine the stimulus within the arena: in two-current choice flumes [29, 30], or using point source delivery [7–9, 31], which complicates the calculation of odor detection threshold and results in different odor exposure duration between fish. Here, we opted for local delivery combined with a rapid and homogeneous distribution of the odor. One drawback of our approach is that it does not allow calculation of a preference index [30, 47, 48]. However, we still observed clear differences in responses to odors with opposite valence, in particular along the vertical dimension of the arena (Fig. 3). In addition, a major advantage was to ensure spatially and temporally reproducible exposure over similar durations, a feature useful for comparing inter-individual differences in olfactory thresholds.

### Metrics for adult zebrafish behavior

Locomotor activity of larval zebrafish is relatively well studied [49–52]; however, the metrics describing adult zebrafish behavior are scarce. Previous studies have used swimming speed and preference index to quantify odor-driven behaviors. Zebrafish react to odors upon detection by an increase or a decrease in swimming speed. However, odors of different valence or ecological significance such as food-related odors and alarm odors can both increase swimming speed [7, 8, 31]. Moreover, the same fish can display biphasic responses to alarm substances, characterized by an alternation of freezing episodes (decreased speed) and bursts of erratic swimming (rapid disorganized swimming with sharp turning angles) [53, 54]. To best describe these temporally and spatially complex adult fish olfactory reactions, it is necessary to use a combination of metrics. Usually, behaviors like erratic swimming are visually quantified by the experimenter [36]. Here, we used an entirely automated analysis to extract metrics classically used in the behavior analysis, such as velocity, burst of acceleration, vertical position in the tank, and amount of freezing. We also used additional parameters such as amount of sharp turns > 90° in order to quantify erratic movements and the patterns of vertical or horizontal swimming in order to examine the direction of swimming trajectory. Interestingly, we found that the five odors eliciting the largest increase in abrupt turns were two decay odors (cadaverine and spermine), two alarm odors (blood and skin extract), and an aversive amino acid (cysteine), indicating that the number of sharp turns could be an appropriate new metric to measure erratic movements in response to aversive stimuli.

### Responses to food odors

Fish detected most feeding odors as indicated by the significant increase in swim velocity and amount of burst swimming in response to four out of seven food odors tested (nucleotides, methionine, arginine, and food extract). Testing each compound separately enabled us to uncover subsets of differing valence within amino acids. Indeed, cysteine and arginine clustered together with the decay-odor spermine and tended to elicit more abrupt turns and bottom diving than other amino acids or the attractive nucleotides resonates with previous findings reporting that larval and adult zebrafish robustly avoid cysteine [20, 33].

### Responses to social odors

Bile acids are thought to be migratory cues guiding sea lampreys to spawning sites [34]. Here, we found that they elicited an increased locomotion, similar to that evoked by food odors in this study and previous reports [7]. Interestingly, urea evoked a very similar response to bile acids and was the closest related odorant in the behavioral space. No strong reaction was found in response to ammonium. Prostaglandin 2α is a steroid hormone produced by ovulating female fish that serves as a reproductive pheromone. It is detected by male fish olfactory system and contributes to the initiation of courtship in goldfish and zebrafish. Previous studies have shown that pgf2α exposure induces moderate behavioral response in male fish tested alone, but dramatic changes when tested in groups [9, 55]. Here, we observed no noticeable response to pgf2α in individually tested fish that were either males or females, confirming the necessity of group testing for this odor. This raises the interesting possibility that multimodal integration of olfactory information with additional sensory cues (vision, touch) is necessary to initiate attraction to pgf2α.

### Responses to alarm odors

Alarm odors that inform about imminent danger are found in insects [56], fish [36], and mammals [57, 58]. Some alarm substances are released by animals when disturbed or attacked, and evoke avoidance or defensive behaviors in conspecifics and sometimes heterospecifics. Prey mammalian species such as the mouse avoid mammalian blood odor [59]. Fish belonging to the Ostariophysi superorder (including zebrafish) display stereotyped anti-predator responses to chemical compounds released by damaged conspecific skin [36]. In agreement with this, we observed a significant increase in freezing and bottom diving in response to zebrafish skin extract. Blood can also be released when conspecifics are injured and was reported to decrease locomotion and to increase the latency to feed in the Nile Tilapia [28]. Extending those findings, we show that low concentrations of conspecific blood (0.01%) elicited strong defensive responses (freezing and bottom diving) in adult zebrafish. Thus, our data suggest that defensive behavior in response to injured conspecifics within shoals could be jointly mediated by damage-released chemical cues present in the blood and skin.

### Responses to decay odors

Decomposition of fish flesh by bacteria releases “death-associated” diamines such as cadaverine [60], which are avoided by adult zebrafish and strongly repulsive in humans [30]. Interestingly, the decay odor spermine clustered with the aversive amino acid cysteine [33]. Both cadaverine and spermine produced significantly more abrupt turns than the water control, similar to responses to negative valence odorants (skin, blood, Fig. 3c), suggesting that the decay odors were perceived as aversive. However, we found that decay odors induce only mild amounts of bottom diving and do not evoke freezing, which is consistent with previous reports [30]. To our knowledge, this is the first zebrafish study that directly compares the responses to decay and alarm odors, which are released by dead or hurt animals. We found that decay odors did not cluster with the potent skin extract and blood alarm odors, confirming that decay odors elicited a response that is qualitatively different from alarm odors in zebrafish. This finding is consistent with the ecology of these odors, given that alarm substances indicate a freshly wounded or killed fish, thus a high probability for an imminent threat, whereas bacteria-mediated production of decay odors takes hours to develop and thus signals a long-gone threat.

### Natural odors versus individual components

Most behaviorally relevant volatile or waterborne odors emitted by preys, predators, and mates are composed of dozens of individual components [8, 31, 61]. In our study including several food-related odors, the food extract elicited the strongest locomotor response (Fig. 3a, b and Additional file 7: Fig. S2A,B) and was segregated in the behavioral space (Fig. 5a, b) compared to stimuli composed of one or two of its individual components, such as methionine and nucleotide mixture. Similarly, skin extract and blood evoked strong defensive responses, yet we did not observe such strong defensive responses to the skin extract component chondroitin sulfate in the same fish. It is unlikely that these differences are due to a lack of detection of the individual chemical components, since they were presented at concentrations higher than the thresholds eliciting responses in zebrafish [7, 31]. Rather, this indicates that individual components of natural feeding and alarm odors do not elicit the full behavioral program. This is consistent with the previous reports showing that in general, animals react stronger to the complete extract present in the natural environment, than to single components of these natural odors. For example, chondroitin sulfate elicits less intense defensive responses than skin extract in zebrafish [31]. Insects like the apple maggot flies or the moth *Manduca sexta* are strongly attracted to crude apple or datura flower extracts, respectively. However, they are only mildly or not attracted to the extract’s chemical components presented alone [61, 62].

### Inter- and intra-individual variability

Despite the increasing amount of studies interested in odor-driven behaviors of zebrafish, few reports whether fish consistently respond to successive presentations of an odor [63], and the variability or robustness of response has not been systematically investigated for a broad range of odorants. We found that less than a third of the odors used here evoked reproducible responses across trials within individual. Interestingly, cadaverine was the only monomolecular odor eliciting strongly consistent response both within and between fish. All other odors eliciting highly consistent responses within fish (bile acids, food, skin extract, and blood) were only weakly correlated across fish. This could be due to the fact that each zebrafish can use consistent but individualized strategies for feeding (bottom versus top feeders) and for defensive behaviors (proactive versus reactive coping styles) [64]. Conversely, chondroitin sulfate, urea, and putrescine induced similar average responses across fish, but were only weakly consistent within fish. This emphasizes that intra- and inter-individual variability parameters describe different aspects of the behavioral responses. Thus, we suggest that odors with low intra-individual and high inter-individual variability are useful stimuli to compare the behavioral and neural responses across fish with different personality traits.

### Neural representation of behaviorally relevant odors

To explore how the stereotyped behavioral responses to odors relate to their internal representations in the olfactory system, we measured the response of the dorsal olfactory bulb to a subset of stimuli at the same behaviorally relevant concentrations. Patterns of neural activity evoked by different categories of odors are coarsely spatially organized in the ventral parts of the zebrafish olfactory bulb [6, 11, 14, 65, 66]. On the dorsal olfactory bulb, we found that bile acids strongly activated the medial parts, in accordance with previous studies in juvenile zebrafish [12]. The amino acid responsive region is located in the ventro-lateral domain [11, 14, 65], which is necessary for the behavioral attraction to amino acids [7]. In line with this, we did not observe strong response to food extract in the dorsal olfactory bulb. We found that skin extract strongly activated neurons located in the antero-lateral parts of the dorsal olfactory bulb, similar to what has been reported in previous studies [13, 31, 32, 66]. Interestingly, we observed that zebrafish blood evoked slightly weaker but very similar activation patterns, which suggest that there might be some shared components between blood and skin extract. Taken together, our results and previous studies suggest that the antero-lateral dorsal olfactory bulb could be specialized in detecting alarm odors that elicit defensive behaviors.

## Conclusion

Our medium-throughput assay provides a low-cost and open setup to reproducibly measure odor-driven behaviors in response to multiple odors in fish. Using this approach enabled us to collect an unprecedented amount of olfactory responses covering the natural stimulus space in adult zebrafish. We confirmed previously described behavioral responses to classically used odorants and also characterized a new powerful alarm substance (blood). We also provide recommendation for future studies to consider the inter- and intra-individual variability of odor-driven behaviors. Future studies will be needed to further explore the neural basis of individual variability in zebrafish olfactory behaviors.

## Methods

### Animals and housing

To allow future comparison with functional imaging studies, the behavioral experiments were carried out in zebrafish *Danio rerio* expressing the fluorescent calcium indicator GCaMP5 pan-neuronally (Tg(elavl3:GCaMP5) [67] in nacre mifta background [68]). A total of 10 fish aged 6–12 months, including three females, were used for the behavioral assays. Fish measured on average 2.5 cm (± 0.16 std) from the tip of the nose to the base of the tail. For calcium imaging experiments, we used three males and one female Tg(elavl3:GCaMP6s) zebrafish [69] in the nacre mifta background and aged 7 months. Fish were kept in 3.5-L tanks in a recirculating fish housing system (Techniplast) at 28 °C under a 14:10-h light/dark cycle. Two weeks before the start of the behavioral experiments, pairs of fish were placed in a 3.5-L tank and isolated from each other by a transparent separator to keep track of individual fish day after day. Fish were fed once in the evening during the behavioral testing period and were thus food-deprived for 18 h before testing.

### Odors

Seventeen odorants, previously documented to activate the olfactory system in a range of aquatic species, and a water control were tested in this study. Single compounds were purchased from Sigma Aldrich (Additional file 1: Table S1). Stock solutions were prepared, kept at − 20 °C, and diluted to final concentration in artificial fish water (AFW; 0.2 g/L marine salt in reverse osmosis water) the morning of the experiment. The final concentration to which the fish were exposed is documented in Additional file 1: Table S1 for each odor. Food odor, blood, chondroitin sulfate, and skin extract were freshly prepared the morning of the experiment. Food odor was prepared by incubating 1 g of commercially available fish food (SDS100, Scientific Fish Food) in 50 mL of AFW for 30 min. The solution was then filtered and further diluted 1:10 in AFW. For blood odor collection, adult nacre fish were rapidly euthanized in ice-cold water, the tail was cut close to the anal orifice, and blood was collected from the dorsal aorta using a 20-μL pipette [70]. Thirty microliters of blood was diluted in 300 mL of ice-cold AFW, filtered, and kept at 4 °C until 1 h before the start of the experiment the same day. Importantly, we made sure to sample blood from the dorsal aorta without touching the skin to avoid contamination by alarm odors released by epidermal club cells. For skin extract, adult nacre fish were rapidly euthanized in ice-cold water and decapitated and the skin was peeled off from the body. The collected skin (0.2 g) was incubated in 1 mL of AFW, mixed and centrifuged at 1300 rpm and 4 °C for 1 h. One milliliter of the supernatant was then dissolved in 300 mL of AFW [12]. Chondroitin sulfate was diluted the morning of the experiment as previously described [31], to avoid damage of this heavy molecule by freezing the stock solutions. Olfactory stimuli for calcium imaging of OB responses consisted in blood, skin extract, taurocholic acid, and food extract diluted in artificial cerebrospinal fluid (ACSF [6]) at the same concentration as for the behavioral assays.

### Behavioral setup

Each fish was allowed to freely swim in a transparent semi-circular vertical arena (horizontal axis = 15 cm; vertical axis = 11.5 cm; width = 3 cm). The arena was made of two transparent petri dishes (Falcon, 15 cm diameter) that were glued together with epoxy resin. After allowing the resin to dry for 2 days, an opening was made at the top using a saw. An outlet tube protected by a fine Nylon mesh was fitted at the bottom of the arena, and an inlet tube consisting in a T-shaped connector (Cole Parmer) was positioned within the arena. Water and odor stimuli were kept in glass bottles positioned on an elevated platform, providing a continuous inflow (90 mL/min), which was measured and adjusted using a flowmeter (Cole Parmer, 65 mm flowmeter with a 3/16″ carboloy float). An outlet tube overflow ensured that the water volume contained in the arena remained constant. To avoid contamination between consecutive stimuli, the tubes were composed of Teflon (Cole Parmer, internal diameter 3.2 mm). The arena was placed within a light-tight enclosure (black hardboard, Thorlab) to isolate the fish from visual interference, and a white LED covered by a diffuser provided homogeneous lighting inside the enclosure. Fish movement was recorded at 10 Hz using a camera (Manta223B, Allied Vision) positioned in front of the arena. Odorant delivery was automatically triggered in synchronization with video acquisition using a Matlab/C++ code and a simple electronic circuit composed of an Arduino Uno (Arduino) and a three-way solenoid valve (Biochem Fluidics). The dynamic of odorant stimulus in the arena was measured using a dye (methylene blue). The day before the experiment started, the fish was habituated to the arena for 3 h, without odorant exposure. Then, at the beginning of each recording day, fish were allowed to habituate to the arena for at least 45 min before initiating recordings. Each trial started by 5 min without odorant; then, the valve was switched open for 1 min allowing the stimulus to be delivered in the arena. Odors were delivered in random order, except zebrafish skin extract and zebrafish blood. Our preliminary recordings showed that zebrafish skin extract and zebrafish blood elicited powerful and long-lasting defensive responses, which is consistent with the prolonged anxiety-like responses observed after alarm substance exposure in other studies [71]. Therefore, these two stimuli were only delivered at the end of a daily recording session. No additional recordings were taken after these, to avoid affecting the response to other odorants. Successive trials were separated by at least 20 min, to allow for complete rinsing of odorant before the next trial started. An average number of 2.0 ± 0.2 replicates per stimulus were collected. The experiments were conducted between 1 and 7 pm in a temperature-controlled room (27 °C). The fish was returned to its home tank and fed every day at the end of the experiment.

### Swim trajectory extraction

The fish position (center of mass) was automatically detected after background subtraction in Matlab (R2016a). Briefly, a background image was obtained by averaging all frames belonging to a given trial. The background was then subtracted from each individual frame. The background-subtracted video was then processed by an erosion/dilation function. In rare cases where the border of the arena or a moving drop of water was detected instead of the fish, a mask was applied to the image to constrain the detection within the arena, and the fish position was re-extracted. To correct for small day-to-day variation in camera’s position compared to the arena, the fish’s position was converted in centimeter and normalized across fish by setting the origin to the bottom left corner of a rectangle circumscribing the arena.

### Behavioral response metrics

We first calculated seven metrics characterizing zebrafish behavior. Instantaneous speed (cm/s) was calculated as the distance between successive positions divided by the recording frequency. Acceleration (cm^2^/s) was the first derivative of speed. Freezing episodes were defined as immobility periods (speed < 0.4 cm/s) that lasted more than 5 s. The number of sudden swimming bursts (acceleration > 1 cm^2^/s) and the amount of sharp changes/turns in swim trajectory (turning angle > 90°) were also quantified. Horizontal and vertical swimming episodes were defined as number of events during which the fish swam with very little deviations (± 10°) from the horizontal and vertical lines, respectively. We then calculated a response metric associated with each behavioral parameter: difference between average value during the 2 min preceding and following the stimulus onset. Positive values indicate an increase of the behavioral metric after stimulus delivery, whereas negative values indicate a decrease. Because we observed biphasic panic responses to alarm odors in most fish (escape first, then freezing), a 4-min time window following the stimulus onset was used for quantifying freezing. These individual metrics were then averaged across replicates of the same odorants to yield a response matrix consisting of 7 metrics × 18 stimuli × 10 fish.

### Analysis of behavioral data

To build the average occupancy maps (Fig. 2), the arena was tilled into 5 mm squares. An occupancy probability was calculated as the number of times the fish was detected within a square, divided by the period length. Occupancy maps were calculated using this method during 4 min before and 4 min during odor exposure. These occupancy maps were then subtracted to obtain a differential occupancy map, which was then averaged per odor and across fish to yield the average maps of areas increasingly explored during odor exposure that are shown in Fig. 2.

To calculate the correlation between behavioral metrics (Fig. 3h), a response vector containing the average value of a given metrics per time bins of 30 s was calculated during the 4 min of odor exposure. The correlation between each pair of metric’s vectors was then calculated using the *pdist* function in Matlab, and averaged across all odors and all fish.

To calculate the variability of behavioral metrics across fish (Fig. 3i), each metric was represented by an 18-element vector composed of the average odor responses per fish. The pairwise vector correlations were calculated between all possible pairs of fish and averaged. To determine if the variability measure was significant for each metric, it was compared with a null distribution of 1000 shuffled values generated by randomly permuting odor responses within each vector and recalculating the pairwise correlations values. The variability of the behavioral metric was considered significant when it was outside the two-sided 95% confidence interval defined by the 2.5th and 97.5th percentiles of the null distribution.

To calculate the trial to trial variability of odor responses (Fig. 4a), each odor replicate was represented by a 7-element vector of all metrics. The pairwise vector correlations were calculated between all possible pairs of replicates and averaged across fish. The significance of trial to trial variability values was calculated for each odor using the same permutation strategy as above.

To calculate the variability of odor responses across fish (Fig. 4b), each odor was represented by a 7-element vector of all behavioral metrics in a given fish. The pairwise vector correlations were calculated between all possible pairs of fish and averaged. The significance of variability of odor responses across fish was calculated for each odor using the same permutation strategy as above.

To calculate the similarity between odors based on the fish behavioral response (Fig. 5), each odor was represented by a 7-element vector composed of the median value of each behavioral metric across fish (Fig. 3a–g, available in Additional file 6: Data S2). The data matrix composed of 18 odors × 7 metrics was *z*-scored to re-scale the behavioral parameters. The Euclidean distance between each pair of odors was then calculated using Matlab’s *pdist* function.

To represent the multidimensional behavioral and neural dataset, we used principal component analysis (PCA). PCA is a method for representing a multidimensional variable space with a few uncorrelated new composite variables, the principal components (PCs) that capture most of the data variability. To build the odors’ representation in the behavioral space (Figs. 5c and 6f), the data matrix composed of 18 odors × 7 metrics was *z*-scored to re-scale the behavioral parameters. Then, the *pca* function of Matlab was used to calculate the first three PCs, the percentage of variance explained, and the loadings of metrics per PC (Fig. 5d–f). To build the odor representation in the neural space (Fig. 6e), the olfactory bulb responses were first standardized across animals by dividing them by the maximal odor response in each fish and pooled together (522 neurons in four fish) and the rest of the procedure was performed as described above.

### Calcium imaging in the nose-attached brain explant

The experiments were conducted in an ex vivo nose-attached brain explant preparation [6, 11, 41, 42], adapted to image the dorsal part of the olfactory bulb. Briefly, three males and one female Tg(elavl3:GCaMP6s) [69] zebrafish were euthanized in ice-cold water. The head was transferred in ice-cold artificial cerebrospinal fluid (ACSF [6]) bubbled with carbogen (95% O_2_/5% CO_2_). The eyes, the jaws, and the dorsal bones covering the telencephalon were carefully removed using forceps. The brain explant was affixed dorsal side up to a small petri dish coated with Sylgard (World Precision Instruments) using tungsten pins. The brain explant was left to equilibrate under the microscope’s ACSF perfusion at room temperature for > 20 min. A mode-locked Ti:Sapphire laser tuned to 920 nm was used to excite GCaMP6s. Olfactory bulb activity was recorded using a two-photon laser scanning microscope (upright with × 16 water immersion objective, Scientifica). Data were collected for twelve optical planes evenly spanning the dorsal olfactory bulb (15 to 180 μm deep). Odor stimuli were delivered to the nose of the brain explant using a computer-controlled valve (Valco-Rheodyne). Stimuli were delivered in three replicates, and the sequence of application was randomized between fish.

### Calcium imaging analysis

Recordings were corrected for movement in Matlab [6]. Recordings were then visually inspected and discarded whenever tissue drift was observed. Stimulus responses were thus calculated based on one to three replicates. Individual neurons were manually segmented, and the corresponding raw fluorescent traces were calculated by averaging the value of all pixels belonging to a given neuron for each time point. The change in fluorescence (%df/f) relative to the pre-stimulus baseline was calculated as follows: %df/f = (Ft − F0)/F0 × 100, where F0 is the averaged 10 s of pre-stimulus fluorescence for each neuron and Ft the fluorescence of a neuron at time *t*. The stimulus response of each neuron was calculated by averaging the %df/f during the 20-s post-stimulus onset. To prepare the odor response maps shown in Fig. 6a, the stimulus response was calculated per pixels for each of the twelve optical planes and averaged across the dorso-ventral axis.

### Statistical analysis

Data were examined for normality of distribution using a Shapiro-Wilk test in Matlab. As none of the metrics described here was normally distributed, difference from the water control condition was detected using a Mann-Whitney *U* test, using a *p* value of 0.05 as threshold for significance. A one-way ANOVA was used to compare the spatial stereotypy of occupancy maps between odors.

## Supplementary information

**Additional file 1: Table S1.** List of odorants used. The odorants used in the study are listed, together with the short names used in the manuscript, concentrations and CAS number of chemicals.

**Additional file 2: Data S1.** Positional information of all fish for all recordings. Column one indicates the fish identity (*n*=10 fish). Column two indicates the odorant delivered. Column three contains the position of the fish center of mass along the horizontal axis (in cm) during 4801 frames (10 frames = 1 second). Column four contains the position of the fish center of mass along the vertical axis (in cm). Odor stimuli are delivered from frame 2401 on. (MAT 17531 kb)

**Additional file 5: Figure S1.** Example traces of change in the seven behavioral metrics in response to selected odors in the same fish (from left to right: food odor, nucleotides, water control, cadaverine, zebrafish blood and zebrafish skin extract). A-G: velocity, burst swimming, number of abrupt turns, horizontal swimming, vertical swimming, time spent freezing and vertical position. Replicates are indicated in black (replicate a) and grey (replicate b). Each behavioral metric was averaged per time bins of 30 seconds and normalized with respect to the baseline period (two minutes before odor delivery). The respective scales are indicated to the right. Horizontal grey dashed lines indicate 0. Vertical grey dashed lines indicate odor onset on each graph.

**Additional file 6: Data S2.** Data related to Figure 3. The median and standard deviation values displayed in Figure 3 are provided for each behavioral metric and each odor. (MAT 30 kb)

**Additional file 7: Figure S2.** Metrics for quantifying behavioral responses of adult zebrafish to ecologically relevant odorants (in connection with Fig. 3). Change in seven behavioral response metrics during the first two minutes following odor delivery: **A)** velocity, **B)** percentage of burst swimming, **C)** number of abrupt turns, **D)** horizontal swimming, **E)** vertical swimming, **F)** freezing, **G)** vertical position in the arena. The filled grey bars indicate significant differences from the water control. Data are represented as median ± standard deviation. Odors are grouped per category. Alarm odors are in red: chondroitin sulfate, zebrafish blood, zebrafish skin extract. Water control is in black. Social-related odors are in blue: prostaglandin 2α, bile acids, urea and ammonium. Decay odors are in magenta: putrescine, spermine, cadaverine. Food-related odors are in green: food extract, nucleotides, histidine, methionine, phenylalanine, cysteine and arginine.

**Additional file 8: Table S2.** Outcome of statistical tests performed in Fig. 3 A-G. *P* values corresponding to all odor – water control pairwise comparisons are displayed here. P values <0.05 are displayed in bold.

**Additional file 9: Figure S3.** Example of individual fish displaying a different behavior. Occupancy maps in response to A) blood and B) bile acids in an individual fish (left panels) and in all other fish (right panels, *n* = 9). The fish swam higher in the tank in response to blood and lower in the tank in response to bile acids, which differed from the responses of the rest of the fish.

**Additional file 10: Figure S4.** Representation of odors in the bi-dimensional spaces composed of behavioral PC1 & PC2 (A), PC2 & PC3 (B) and PC1 & PC3 (C). Grey lines indicate the distance of each odor to the water control (filled black dot). Food-related odors (food extract, histidine, nucleotides, methionine, phenylalanine, cysteine and arginine) are represented by green circles. Social-related odors (bile acids, prostaglandin 2α, urea and ammonium) are represented by blue squares. Decay odors (putrescine, spermine, cadaverine) are represented by magenta asterisks. Alarm odors (chondroitin sulfate, zebrafish blood, zebrafish skin extract) are represented by red crosses.

**Additional file 11: Data S3.** Olfactory bulb responses to odors in connection with Figure 6. A) Average olfactory bulb responses to zebrafish blood, zebrafish skin extract, food odor, bile acid and saline control in all fish (*n*=4) used in Figure 6. B) Time course of odor responses for the three example neurons displayed in Figure 6C. C) Individual correlation matrices (n=4 fish) representing the average pairwise similarity between multi-neuronal odor representations used in Figure 6D. (MAT 281 kb)

## Data Availability

All data generated or analyzed during this study are included in this published article and its supplementary information files (Additional files: Data S1-3).
