# Supplementary figures and images for: Stimulus-specific behavioral responses of zebrafish to a large range of odors exhibit individual variability
Source: BMC Biol. 2020 Jun 15;18:66. doi: 10.1186/s12915-020-00801-8 (PMC7296676; doi:10.1186/s12915-020-00801-8)

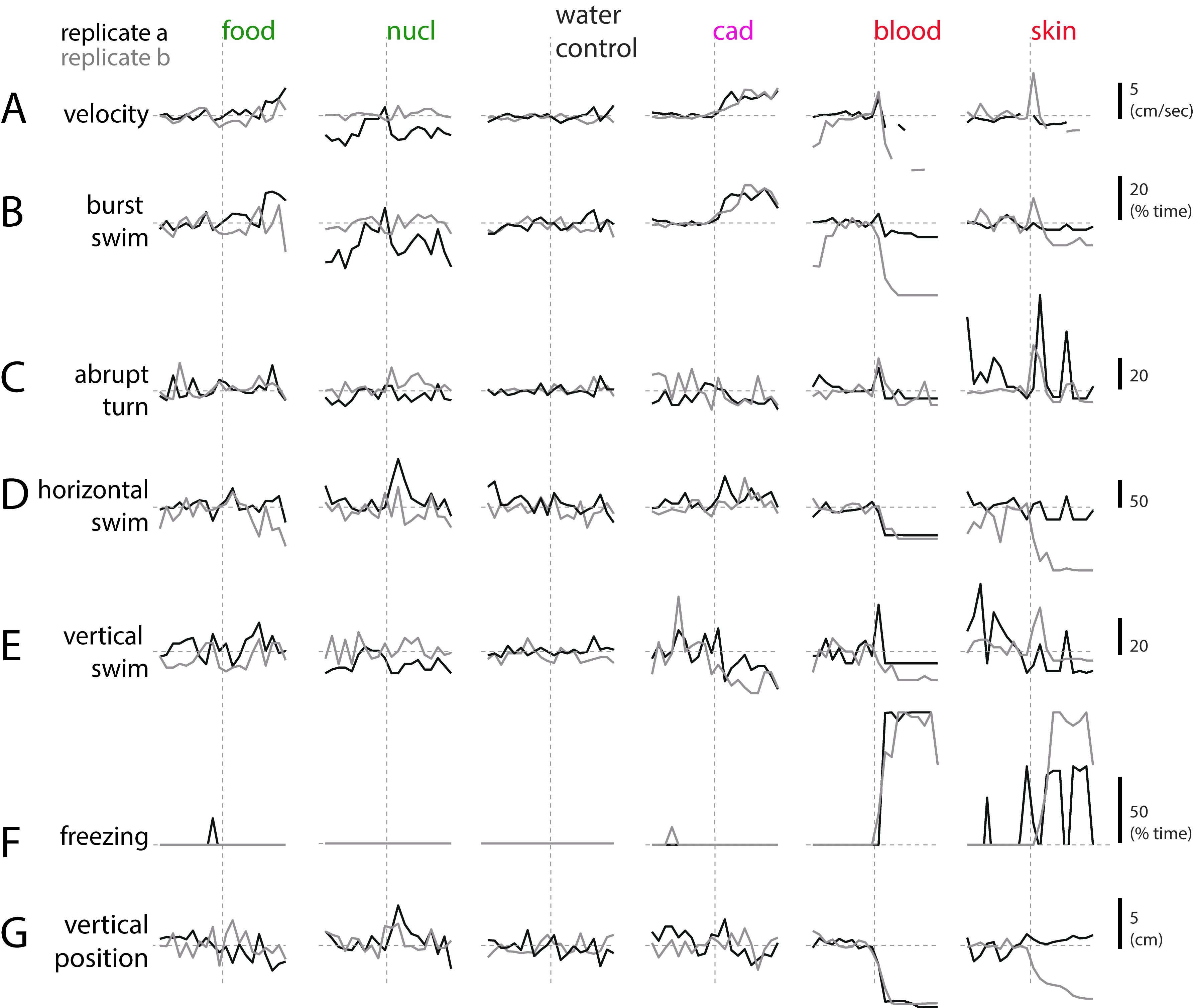

Supplement: Supplementary file 5 — Additional file 5: Figure S1. Example traces of change in the seven behavioral metrics in response to selected odors in the same fish (from left to right: food odor, nucleotides, water control, cadaverine, zebrafish blood and zebrafish skin extract). A-G: velocity, burst swimming, number of abrupt turns, horizontal swimming, vertical swimming, time spent freezing and vertical position. Replicates are indicated in black (replicate a) and grey (replicate b). Each behavioral metric was averaged per time bins of 30 seconds and normalized with respect to the baseline period (two minutes before odor delivery). The respective scales are indicated to the right. Horizontal grey dashed lines indicate 0. Vertical grey dashed lines indicate odor onset on each graph. [file 12915_2020_801_MOESM5_ESM.jpg]

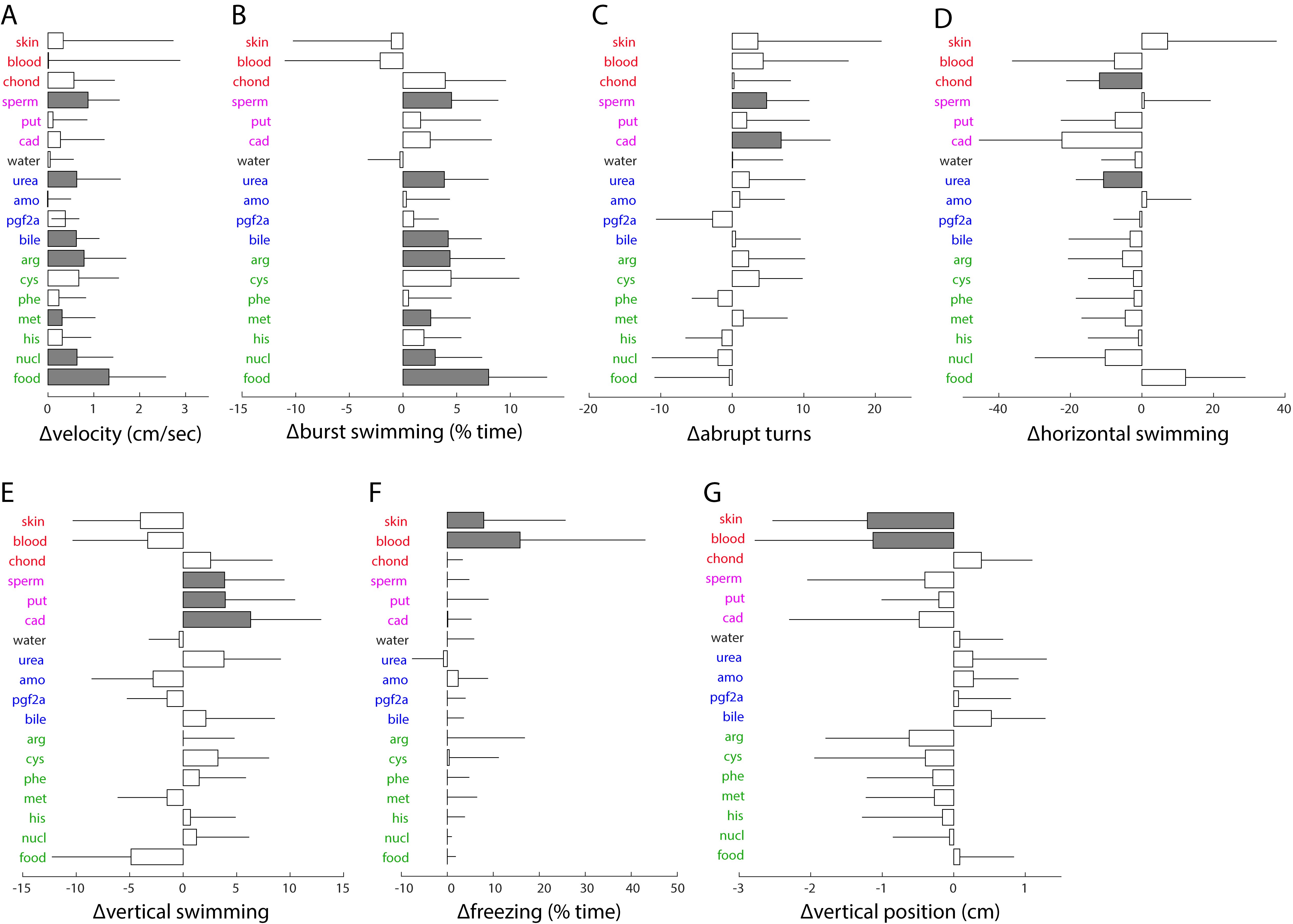

Supplement: Supplementary file 7 — Additional file 7: Figure S2. Metrics for quantifying behavioral responses of adult zebrafish to ecologically relevant odorants (in connection with Fig. 3). Change in seven behavioral response metrics during the first two minutes following odor delivery: A) velocity, B) percentage of burst swimming, C) number of abrupt turns, D) horizontal swimming, E) vertical swimming, F) freezing, G) vertical position in the arena. The filled grey bars indicate significant differences from the water control. Data are represented as median ± standard deviation. Odors are grouped per category. Alarm odors are in red: chondroitin sulfate, zebrafish blood, zebrafish skin extract. Water control is in black. Social-related odors are in blue: prostaglandin 2α, bile acids, urea and ammonium. Decay odors are in magenta: putrescine, spermine, cadaverine. Food-related odors are in green: food extract, nucleotides, histidine, methionine, phenylalanine, cysteine and arginine. [file 12915_2020_801_MOESM7_ESM.jpg]

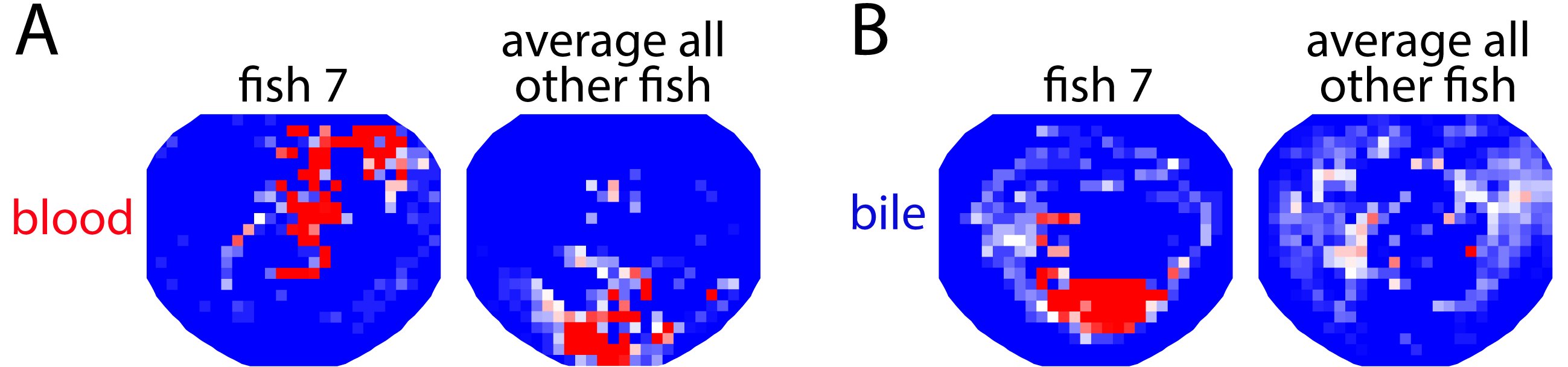

Supplement: Supplementary file 9 — Additional file 9: Figure S3. Example of individual fish displaying a different behavior. Occupancy maps in response to A) blood and B) bile acids in an individual fish (left panels) and in all other fish (right panels, n = 9). The fish swam higher in the tank in response to blood and lower in the tank in response to bile acids, which differed from the responses of the rest of the fish. [file 12915_2020_801_MOESM9_ESM.jpg]

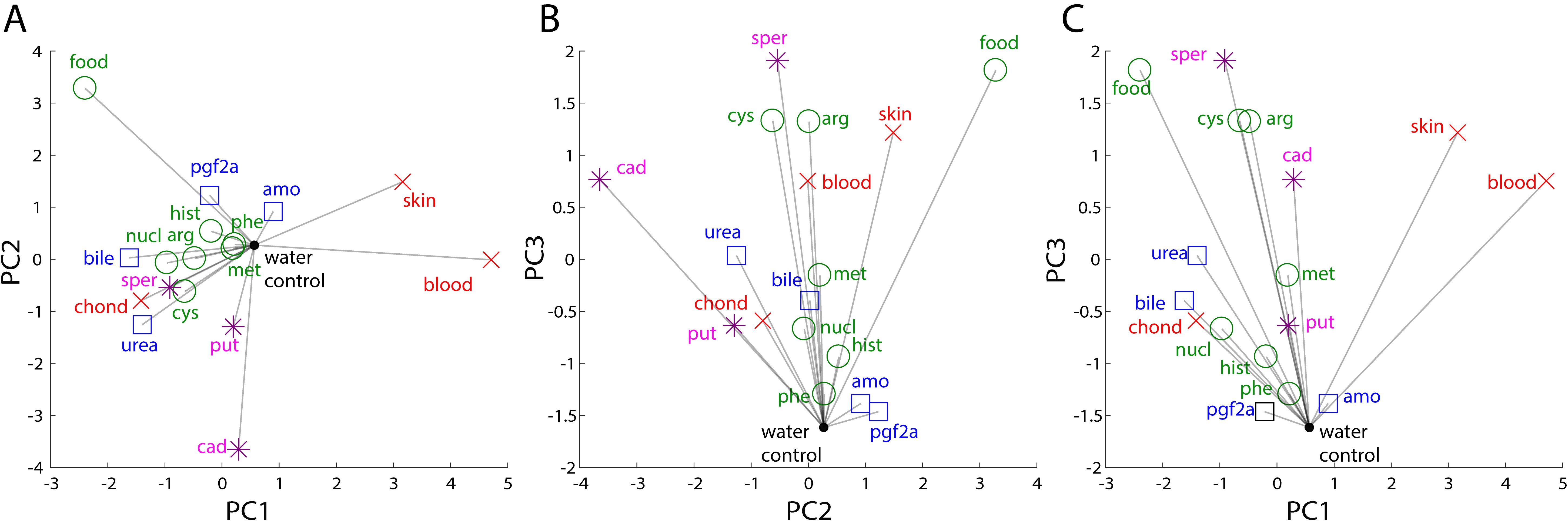

Supplement: Supplementary file 10 — Additional file 10: Figure S4. Representation of odors in the bi-dimensional spaces composed of behavioral PC1 & PC2 (A), PC2 & PC3 (B) and PC1 & PC3 (C). Grey lines indicate the distance of each odor to the water control (filled black dot). Food-related odors (food extract, histidine, nucleotides, methionine, phenylalanine, cysteine and arginine) are represented by green circles. Social-related odors (bile acids, prostaglandin 2α, urea and ammonium) are represented by blue squares. Decay odors (putrescine, spermine, cadaverine) are represented by magenta asterisks. Alarm odors (chondroitin sulfate, zebrafish blood, zebrafish skin extract) are represented by red crosses. [file 12915_2020_801_MOESM10_ESM.jpg]
